# Possible Implications of Obesity-Primed Microglia that Could Contribute to Stroke-Associated Damage

**DOI:** 10.1007/s10571-023-01329-5

**Published:** 2023-03-20

**Authors:** Ricardo Jair Ramírez-Carreto, Yesica María Rodríguez-Cortés, Haydee Torres-Guerrero, Anahí Chavarría

**Affiliations:** Unidad de Investigación en Medicina Experimental, Facultad de Medicina, Universidad Nacional Autónoma de México, Mexico City, Mexico

**Keywords:** Brain damage, Low-grade chronic inflammation, Microglial polarization, Neuroinflammation, Obesity, Stroke

## Abstract

Microglia, the resident macrophages of the central nervous system, are essential players during physiological and pathological processes. Although they participate in synaptic pruning and maintenance of neuronal circuits, microglia are mainly studied by their activity modulating inflammatory environment and adapting their phenotype and mechanisms to insults detected in the brain parenchyma. Changes in microglial phenotypes are reflected in their morphology, membrane markers, and secreted substances, stimulating neighbor glia and leading their responses to control stimuli. Understanding how microglia react in various microenvironments, such as chronic inflammation, made it possible to establish therapeutic windows and identify synergic interactions with acute damage events like stroke. Obesity is a low-grade chronic inflammatory state that gradually affects the central nervous system, promoting neuroinflammation development. Obese patients have the worst prognosis when they suffer a cerebral infarction due to basal neuroinflammation, then obesity-induced neuroinflammation could promote the priming of microglial cells and favor its neurotoxic response, potentially worsening patients’ prognosis. This review discusses the main microglia findings in the obesity context during the course and resolution of cerebral infarction, involving the temporality of the phenotype changes and balance of pro- and anti-inflammatory responses, which is lost in the swollen brain of an obese subject.

**Graphical Abstract:**

Obesity enhances proinflammatory responses during a stroke. Obesity-induced systemic inflammation promotes microglial M_1_ polarization and priming, which enhances stroke-associated damage, increasing M_1_ and decreasing M_2_ responses.

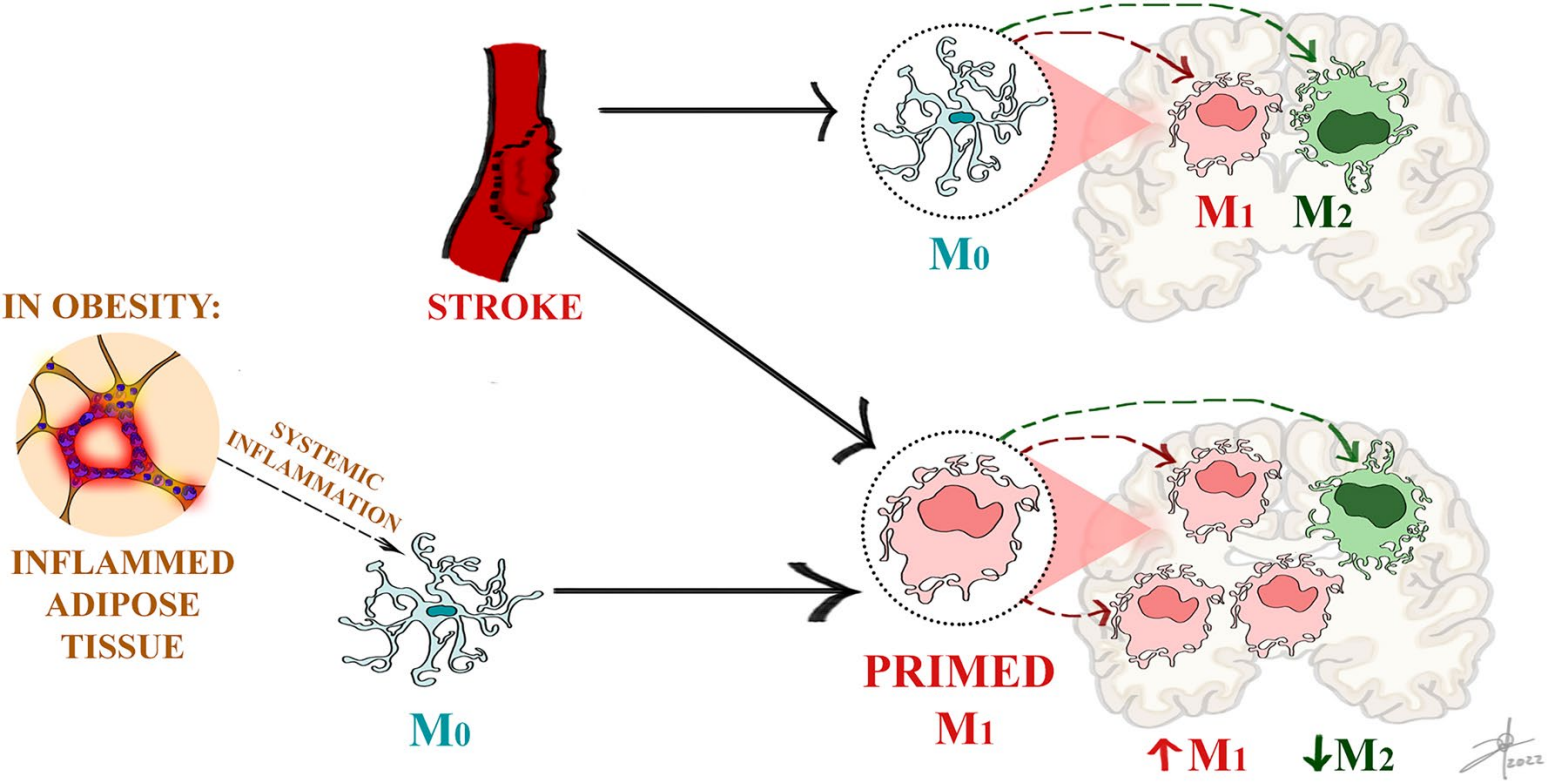

## Introduction

Nowadays, urban lifestyle, exposure to hypercaloric food and drinks, and lack of physical activity contribute to overweight and obesity development in the general population worldwide (De Lorenzo et al. 2016). According to the World Health Organization (WHO), until 2016, more than 1.9 billion adults worldwide were overweight, and 650 million were obese. Now, obesity is considered a pandemic and public health problem. The WHO defines overweight as a condition in which body mass index (BMI) is greater than or equal to 25, and obesity when the BMI is greater than or equal to 30 (World Health Organization 2017). Obesity is associated with multiple comorbidities due to its impact on physiology span and promotion of chronic inflammation in the adipose tissue (Michailidou et al. 2022); this local inflammation gradually becomes systemic and affects many tissues and organs, including the central nervous system (CNS) (Alexaki 2021). In this region, there are different cell types, highlighting neurons and glia, mainly consisting of microglia, astrocytes, and oligodendrocyte lineage cells. Microglia is the primary immunocompetent glia member, which can respond to peripheral inflammatory signals and drive neuroinflammation (Cunningham 2013; Alexaki 2021). Systemic chronic inflammation and metabolic changes can influence the microglial response, inducing a primed proinflammatory state that increases the risk of neuroinflammation-related diseases and enhanced damage secondary to acute traumatic and ischemic CNS events (Qin et al. 2019; Kluge et al. 2019; Alexaki 2021). In addition, obesity is also a risk factor for developing neuropathologies such as Alzheimer’s disease (AD) (Lloret et al. 2019), Parkinson’s disease (PD) (Chen et al. 2004), or even ischemia (O’Donnell et al. 2010; Pandian et al. 2018). Likewise, animal studies demonstrated that obesity-derived low-grade systemic chronic inflammation exacerbates damage in some neurological disorders like dopaminergic degeneration in PD (Kao et al. 2020) or elevates neuroinflammation in enhanced depression-like behavior (Wang et al. 2022).

Currently, the association between obesity, metabolic dysfunction, neuroinflammation, and mortality in stroke patients is not well-established; however, some cohort studies have shown a correlation between body mass index and abdominal obesity with ischemic and hemorrhagic stroke (Shiozawa et al. 2021; Akyea et al. 2021; Jaakonmäki et al. 2022). For all these reasons, this review aims to analyze how obesity-related systemic inflammation can affect microglial response in the CNS, prime them, leading to increased stroke-associated neuronal damage secondary to obesity and chronic neuroinflammation, linking them as risk factors that decrease survival and worsening patient’s prognosis as correlated in epidemiological studies (Shiozawa et al. 2021; Akyea et al. 2021; Jaakonmäki et al. 2022). Herein, we review the roles of microglia and their functional interactions and regulatory mechanisms in obesity and stroke, and how obesity-primed microglia could enhance stroke-associated damage.

## Generalities of Microglial Cells

Microglia are resident macrophages of CNS in mammals, derived from yolk sac myeloid progenitors during neurodevelopmental stages, colonize the brain early in brain development and establish in the brain parenchyma. In the adult stage, they represent about 10% of the CNS population (Lawson et al. 1990; Jurga et al. 2020) and 5%–20% of glial cells (Polazzi and Monti 2010; von Bartheld et al. 2016; Zhang et al. 2018). These resident brain immune cells are divergent from other peripherally immunocompetent cell populations, such as infiltrated, perivascular, and borders-associated macrophages due to their ontogeny from the yolk sac and autorenewal capacity, about 28% per year, with a lifespan of 4.2 years, totally independent from bone marrow (Réu et al. 2017). However, their precise discrimination is difficult due to their myeloid origin when analyzed in situ.

Microglia are involved in several physiological processes such as synaptic pruning, learning, memory, neurogenesis, and neuronal connectivity through the secretion of neurotrophic factors, including brain-derived neurotrophic factor (BDNF), insulin-like growth factor 1 (IGF-1), and glial cell-derived neurotrophic factor (GDNF), among others (De Biase et al. 2017; Zhang et al. 2018; Hammond et al. 2018). This communication between neurons and microglia maintains an optimal neural network, drives the formation of functional neuronal connections, and regulates neuronal survival, considered a homeostatic neuronal microenvironment (Wang and Li 2021). Nevertheless, microglia are mainly studied by their immunological surveillance functions and defense mechanisms against pathogens and insults (Uriarte Huarte et al. 2021). Under normal conditions, adult microglia have been described morphologically as compact cells with a rounded and short body, with large and fine processes. In response to the peripheral inflammation, external signals, and circulating mediators, microglia undergo rapid morphological adaptations and transitions from their ramified, homeostatic form; to a motile, amoeboid-shaped cell.

When a ramified microglia detects changes in the neuronal microenvironment recognizing neurotoxic, inflammatory, or dysfunctional signals, these cells can extend and retract their processes to migrate to the injured site (McConnell and Mishra 2022) and also initiate intracellular cascade reactions to make them undergo to an activated state, which is considered an intermediate form between the ramified and ameboid state (Parakalan et al. 2012). In this form, microglia retract and thicken their branches, expressing molecular profiles to respond and adapt to environmental needs involved in a critical role by regulating reactive astrogliosis, phagocytosing debris, and signaling to peripheral immune cells (McConnell and Mishra 2022). At last, microglia turn into a fully active ameboid form -morphologically similar to peripheral macrophages- and get macrophages-like polarization states as M_1_ and M_2_ (Bohatschek et al. 2001; Parakalan et al. 2012; Cai et al. 2014).

Depending on the context of the brain parenchyma, microglial cells can shift into active phenotypes to counteract changes in the neuronal microenvironment, such as potential neurotoxic insults, dysfunctional synapses, or physical disruption of brain parenchyma by cleaning cell debris, killing pathogens, healing and repair tissue or regulate neurofunctional networks (Full review by Hammond et al. 2018). Classifying microglia in either an M_1_ or M_2_ polarized state may be an oversimplification due to their vast repertoire of molecular responses, states, and functions, which are only defined using transcriptomics and proteomics (Paolicelli et al. 2022). However, nowadays, the macrophage-like polarization state model helps understand microglial roles during the development and progression of ischemic stroke (Yu et al. 2022; Chen et al. 2022). Hence this work will use the M_1_/M_2_ model to approach the interaction between obesity-related systemic inflammation, microglia priming, and stroke.

Microglia can dynamically change to multiple polarization states, but most described are M_1_-like and M_2_-like phenotypes; this represents a continuous assortment of different activation phenotypes that can coexist (Wendimu and Hooks 2022). The presence of multiple activation phenotypes for microglia is associated with the homeostatic state and the physiological and pathological changes in the tissue. Microglia polarization states have been characterized as classic activation M_1_, alternative activation M_2*a*_, alternative activation M_2*b*_, or acquired deactivation M_2*c*_ (Walker and Lue 2015). One limitation of the M_1_ or M_2_ phenotyping is that it excludes microglia undergoing cell division (M_3_) as a response to macrophage colony-stimulating factor CSF-1 or IL-34. This cell division by microglia is a relevant feature in pathology-rich areas where damaged cells need to be replaced (Schwabenland et al. 2021).

Their differentiation depends on the activation of specific pathways, such as PI3K/Akt/mTOR or JAK1, which contribute to the morphological changes of microglial cells (Akhmetzyanova et al. 2019). The most found phenotypes in stroke are described below, emphasizing the phenotypes associated with disease (Candlish and Hefendehl 2021).

Animals and humans have different microglial populations, which must be considered. Differences in microbiology, genetics and even location (mice keep most of their microglia in gray matter, whereas humans keep them in white matter) (Agnieszka et al. 2020) make it challenging to define neuroinflammation in humans with animal models accurately. Nevertheless, there are shared markers among different species, such as the transmembranal protein 119 (TMEM119); this protein is abundant in the prenatal periods (Bennett et al. 2016). Hence the following sections will refer to microglial markers supported by rodent models to understand the vast repertoire of microglial molecular sets.

### Microglia M_0_ and Discrimination from Peripheral Macrophages

**Fig. 1:**
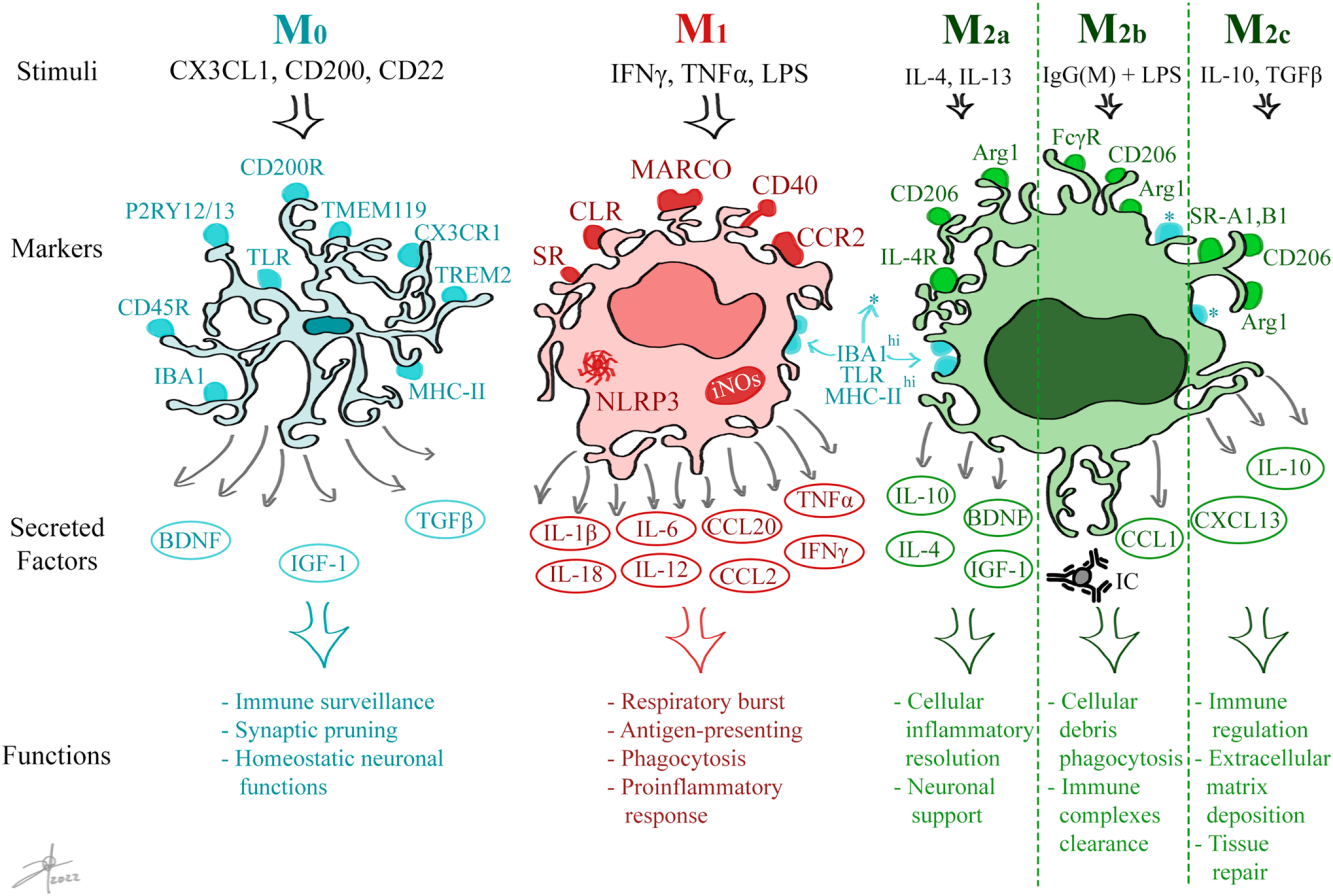
Microglial activation states, markers, and functions. Microglia are brain immune cells that can activate into three different states depending on the stimuli and tissue needs. In the M0 activation state, microglia, with neurons and other glial cells, maintain homeostatic functions by secreting neurotrophic factors and anti-inflammatory cytokines while surveilling from potentially neurotoxic agents. The M1 activation state exerts proinflammatory functions. The M2 activation state can be further subdivided into M2a, M2b, and M2c subsets. M2a microglia resolve inflammation by secreting neurotrophic factors and anti-inflammatory cytokines. M2b is a phagocytic subset that clears immune complexes and cellular debris. M2c microglia repair damaged tissue and regulate cellular immune responses by secreting anti-inflammatory cytokines. Abbreviations: arginase 1 (Arg1), brain-derived neurotrophic factor (BDNF), cluster of differentiation (CD), C-type lectin receptors (CLR), gamma Fc receptor (FcγR), ionized calcium-binding adapter 1 (IBA1), immune complexes (IC), interferon *γ* (IFN*γ*), insulin-like growth factor 1 (IGF-1), immunoglobulin G and M (IgG(M)), interleukin (IL), IL-4 receptor (IL-4R), inducible nitric oxide synthase (iNOs), macrophage receptor with collagenous structure (MARCO), major histocompatibility complex II (MHC-II), purinergic class 2 Y12 and Y13 receptors (P2RY12/13), LPS scavenger receptors (SR), A1 and B1 class scavenger receptors (SR-A1, B1), transforming growth factor β (TGFβ), toll-like receptor (TLR), transmembranal protein 119 (TMEM119), tumor necrosis factor α (TNFα), triggering receptor expressed on myeloid cells 2 (TREM2)

Microglia M_0_ is in an active surveillant phenotype characterized by basal expressed surface markers such as the cluster of differentiation (CD)11*b*, F4/80, CX3CR1, P_2_Y_12_R, and TMEM119 (Butovsky et al. 2014; Amadio et al. 2014; Orihuela et al. 2016; Bennett et al. 2016), intracellular proteins as ionized calcium-binding adapter 1 (IBA1), and vimentin (Butovsky and Weiner 2018; Jurga et al. 2020), and transcription factors as SALL1, and Pu.1 (Yeh and Ikezu 2019) (Fig. 1). A critical feature is that most of these molecules are shared with macrophage populations, so profile markers are needed to discriminate them and characterize exclusive microglial participation in physiological processes. Some studies suggest that using quantitative markers can help with the dissertation between resident microglia, which have CD11b^+^/CD206^low/−^/CD163^−^ profile, and peripheral macrophages that present a CD11b^+^/CD206^high^/CD163^+^ profile (Fig. 1) (Ford et al. 1995; Grabert et al. 2016; Jurga et al. 2020). Microglia are strictly regulated by other glia and neurons to maintain a non-reactive profile, which can be neurotoxic, by binding to inhibiting receptors CX3CR1, CD200R, neurotransmitters receptors, and CD45R (Ransohoff and Cardona 2010; Orihuela et al. 2016). Also, an enriched transforming growth factor *β* (TGF*β*) microenvironment is needed to keep microglia attenuated (Butovsky et al. 2014), then M_0_ microglia provides a neurotrophic factors microenvironment (BDNF and IGF-1) to maintain homeostatic neuronal functions (Franco and Fernández-Suárez 2015).

### Microglia M_1_ and Proinflammatory Response to Insults

When microglia detect proinflammatory, pathogenic, or homeostatic disrupting signals, they can turn over to a reactive ameboid phenotype, changing their morphology to a macrophage-like form, described as a rounded body cell, with short and thick pseudopodia, that enables microglia to go towards the danger area, release cytotoxic and proinflammatory molecules, and perform phagocytosis (Dihné et al. 2001; Jurga et al. 2020).

Microglia can recognize any changes in the neuronal microenvironment which implicate potential danger to neurons. These signals can be detected by surface proteins that recognize common pathogen patterns (PAMPs), such as lipopolysaccharide (LPS), or cellular damage signals (DAMPs), like membrane lipids or adenosine triphosphate (ATP), using pattern recognition receptors (PRR), mainly Toll-like receptors (TLR) and NOD-like Receptors (NLR), but also purinergic-, Fc-, complement-, scavenger-, chemokine-, and cytokine- receptors, and other recognition proteins that can activate microglia to polarize to a classically activated state (Orihuela et al. 2016; Butovsky and Weiner 2018; Jurga et al. 2020). When microglia cells detect subtle changes, generally directed by LPS or interferon *γ* (IFN*γ*), they trigger an inflammatory state which is strictly regulated and silenced over time. In this state, microglia secrete cytokines to communicate with neighbor cells, alerting and influencing their functions. This state is known as M1 and was named for its analogy with the macrophage activation state, which enhances antigen presentation, produces and secretes proinflammatory cytokines IL-1*β*, IL-6, IL-12, IL-18, tumor necrosis factor ⍺ (TNF*⍺*), IFN*γ*, and chemokines CCL2, CCL5, CCL20, CXCL1, CXCL9, among others (Orihuela et al. 2016; Liu et al. 2018; Jurga et al. 2020). In addition, microglia cells promote the respiratory burst by producing reactive oxygen (ROS) and nitrogen (RNS) species using NADPH oxidase and iNOS systems, respectively (Bordt and Polster 2014; Ghosh et al. 2018). It also contributes to glial scar formation and regulates synaptic pruning and phagocytosis (Fig. 1) (Nakagawa and Chiba 2014; Akhmetzyanova et al. 2019).

The inflammatory response of M_1_ is necessary for damage control and eradication, as occurs in acute neuroinflammation, and classically activated microglia show an enhanced expression of CD40, MHC-II molecules, CD16, CD32, CD86, MMP-9, and macrophage receptor with collagenous structure (MARCO) (Fig. 1) (Orihuela et al. 2016; Paolicelli et al. 2022).

### Microglia M_2_ and Anti-Inflammatory/Tissue Repair Subsets

In contrast to the M_1_ state, microglia can be stimulated by anti-inflammatory factors and alternatively be activated to perform neuroprotective, neuroregenerative, anti-inflammatory, and tissue reparative responses (Franco and Fernández-Suárez 2015). This state is known as M_2_ and has a broad spectrum of responses, so some studies and research groups have divided M_2_ into three different subsets to its functional role: M_2a_, M_2b_, and M_2c_ (Almolda et al. 2015; Orihuela et al. 2016; Zhang et al. 2018) (Fig. 1).

M_2*a*_ subset is induced by the exposure of microglia to anti-inflammatory cytokines IL-4 and IL-13 and contributes to the T_H2_-like responses and repair of damaged tissue by expressing anti-inflammatory cytokines and neurotrophic factors. On the other hand, microglia polarize to an M_2*b*_ profile by binding agonists to the IL-1 receptor antagonist (IL-1Ra), TLR4, or Fc receptors, performing enhanced phagocytosis functions, acting as cell debris and immune complexes cleaner; they also secrete anti-inflammatory cytokines. At last, exposure to glucocorticoids, IL-10, and TGFβ promote the M_2*c*_ phenotype, which focuses its mechanisms on immunoregulation, matrix deposition, and tissue remodeling by enhancing arginase 1 (ARG1) activity, secreting chemokine CXCL13 and overregulating scavenger receptors A1 and B1 (SR-A1, SR-B1) (Fig. 1) (Martinez et al. 2008; Franco and Fernández-Suárez 2015; Orihuela et al. 2016; Zhang et al. 2018).

Altogether, the microglia M_2_ phenotype is considered anti-inflammatory and upregulates surface markers such as CD206 and CD163, promotes tissue regeneration, waste removal, neurogenesis, remyelination, and neurite outgrowth through the secretion of IGF-1, BDNF, and miR-124-3P; it also induces autophagy and blood–brain barrier (BBB) repair through the secretion of anti-inflammatory cytokines and growth factors (Franco and Fernández-Suárez 2015; Orihuela et al. 2016; Akhmetzyanova et al. 2019; Wan et al. 2022; Paolicelli et al. 2022).

### Microglia in Disease and Priming

Due to their multiple functions, critical roles, and plasticity in the homeostatic brain, microglia have been implicated in various neurological disorders (Nakagawa and Chiba 2014; Zhang et al. 2018). Multiple studies have described the role of microglia in different neuropathologic disorders/conditions (Streit et al. 2004). For example, amyloid-beta plaques have a chemotactic effect on microglia. The plaque size, especially those positive for ThioS, attract microglial cells and is independent of neuronal damage (Serrano-Pozo et al. 2013). In PD, the inhibition of microglia activation using NLY01 -a glucagon-like peptide-1 receptor (GLP-1R) agonist- modifies the transformation of A_1_ astrocytes contributing to the inflammatory event, which generates a neuroprotective response (Yun et al. 2018). In pain models, mesenchymal stem cells that produce gene 6 (TSG-6) protein negatively regulate the TLR2/MyD88/NF-kB signaling, reducing the production of microglial proinflammatory cytokines, generating neuroprotection and relief of neuropathic pain (Yang et al. 2020). The microglial response lasts up to two months following the infarction, even at a different site of the initial lesion, influencing penumbral regions and causing severe neurodegeneration elsewhere (Kluge et al. 2019).

In some contexts, a discrete disruption in homeostasis induces a cascade of adaptive responses in glia, particularly in microglia, involving biochemical, morphological, and functional changes associated with the production of basal cytokines and secondary mediators that influence neuronal networks, cognition, and behavior. This response is named “priming” and generally represents a beneficial response, but it may become maladaptive when microglia integrity and function are compromised (Norden et al. 2015). Macrophages and microglia can develop a primed proinflammatory profile consisting of an amplified response mRNA expression, protein translation, and morphological changing when exposed to stress (Niraula et al. 2017), aging (Norden and Godbout 2013; Niraula et al. 2017), chronic systemic inflammation (Chouhan et al. 2022), and obesity (Alexaki 2021). Due to this maladaptive response, microglia exacerbate their inflammatory response when an acute event happens, such as traumatic brain injury (TBI) (Lifshitz et al. 2007), pathogen-associated neuroinflammation (Furr and Marriott 2012; Lima et al. 2022), and stroke (Qin et al. 2019), among others. As a result of this stage, microglia show a high rate of proliferation after being primed and present impairments in regulatory systems, circumstances that make microglia resistant to negative feedback and functionally compromised, enhancing resultant damage, and worsening the patient’s prognosis (Norden et al. 2015).

In related neurological diseases, a positive severity relationship was observed in patients who were obese previously to the neurological disease development. Obesity rates were higher in patients with refractory than non-refractory epilepsy (Janousek et al. 2013) and correlated as a potential factor for developing a drug-resistant variant (Chen et al. 2021). In TBI, epidemiological data suggest that obese patients develop more complications and higher mortality than lean patients, which is also enhanced by age (Brown et al. 2006). Some studies reported learning and memory deficits in obese patients compared to non-obese (Elias et al. 2003, 2005). Other studies confirmed the association of obesity with behavioral declines in executive function (Gunstad et al. 2007). A study with 6582 participants in England showed that people over 50 with increased BMI or abnormal obesity were associated with increased dementia incidence (Ma et al. 2020). Obesity is a risk factor for AD development as the increase in BMI affects some brain structures like cortical areas, and weight loss reverses brain atrophy, so authors refer to the “Obesity paradox” as a bias explained by reverse causation (Pegueroles et al. 2018). In addition, visceral obesity favors AD development through tissue injury, oxidative stress, leptin resistance, inflammatory changes, glutamate excitotoxicity, and hypoadiponectinemia that collectively trigger neuroinflammation and amyloid β deposits (Lloret et al. 2019; Al-Kuraishy et al. 2022). Obesity animal models also support these pathological synergic findings; for example, diet-induced obesity in rodents can synergize with the TBI model by decreasing hippocampal plasticity and learning (Wu et al. 2003). Another study showed that diet-induced obesity and insulin resistance decrease spatial learning skills in rats (Stranahan et al. 2008). In addition, a high-fat diet (HFD) increases the amyloid burden and decreases cognitive behavior in an AD transgenic model (Fewlass et al. 2004). What kind of mechanisms are associated with this synergic effect? As discussed, systemic inflammation can promote microglia priming, and this activation state also synergizes the bad prognostic in some pathologic conditions.

## Obesity Induces Changes in Microglial Cells

The white adipose tissue (WAT) is considered an endocrine organ due to its adaptability to the body’s energy needs and its production of several proteins that regulate hunger and satiety, including hormones such as leptin and insulin, and cytokines; these factors are known as adipokines and perform several different functions (Lago et al. 2007).

### Cytokines and Immune cells Dynamics in the Adipose Tissue

**Fig. 2:**
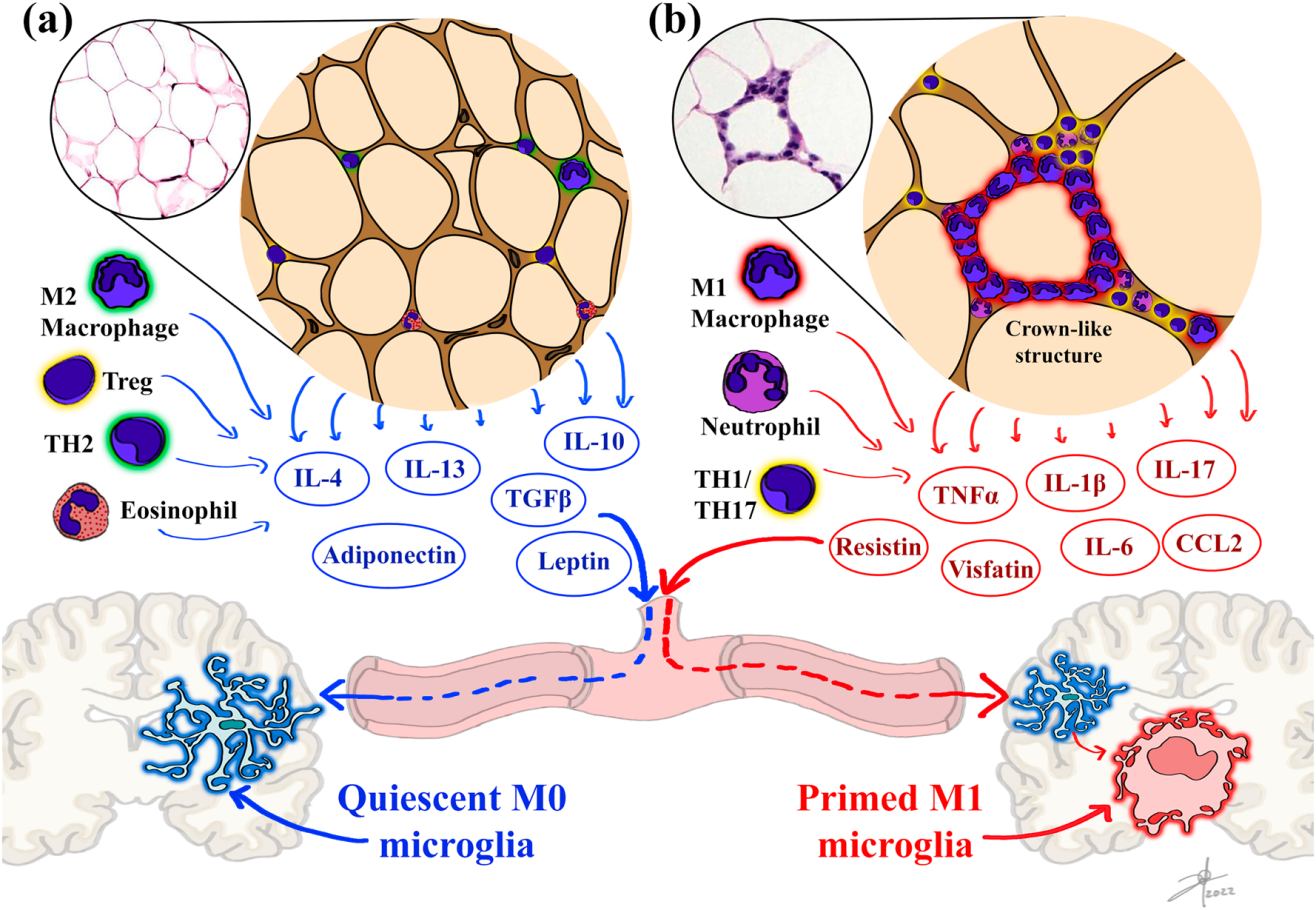
Inflammatory dynamics in the adipose tissue and its consequences in CNS. Adipose tissue’s endocrine and immune regulatory functions change during obesity. **a** Lean adipose tissue contains adipocytes and immune regulatory cells as M_2_ macrophages, T_reg_, T_H2_ lymphocytes, and eosinophils, which secrete homeostatic adipokines preserving microglia M_0_ phenotype. **b** Obese adipose tissue contains hypertrophic and hyperplasic adipocytes that recruit and polarize proinflammatory immune cells as neutrophils, T_H1_ and T_H17_ lymphocytes, and M_1_ macrophages, which form crown-like necrotic proinflammatory structures. Together these cells secrete proinflammatory adipokines inducing M_1_ microglia priming. Abbreviations: interleukin (IL), tumor necrosis factor *⍺* (TNF*⍺*)

Under healthy homeostatic physiological conditions, lean WAT maintains sensitivity toward hormone-related signals by releasing anti-inflammatory cytokines such as TGFβ and IL-10 by the resident M_2_ macrophages and T_reg_ cells (Kawai et al. 2021), IL-4 and IL-13 by eosinophils (Wu et al. 2011), IL-4, IL-5, IL-6, IL-10, and IL-13 by T_H2_ cells (Kawai et al. 2021), and IL-1Ra together with many hormones as leptin, adiponectin, resistin, visfatin, apelin, omentin, among others by adipocytes (Fully reviewed by (Lago et al. 2007)) favoring metabolic functions and signaling (Fig. 2a). In contrast, during obesity, the excess of macronutrients in the WAT influences adipocytes to become hyperplasic and hypertrophic, this event is accompanied by infiltration of peripheral immune cells, such as neutrophils, T_H1,_ and T_H17_ cells, and macrophages polarized into an M_1_ profile (Ellulu et al. 2017; Unamuno et al. 2018). Along with these immune cells, adipocytes acquire an inflammatory profile producing and releasing a variety of inflammatory molecules such as TNF*α*, IL-1*β*, IL-6, IL-17, and CCL2, promoting cell recruitment and enhancing the proinflammatory response (Fig. 2b) (Bertola et al. 2012; Ellulu et al. 2017). Inflamed adipocytes and peripheral immune cells secrete proinflammatory cytokines, locally and systemically, then the WAT can be considered an immune and secretory organ (Kawai et al. 2021). Also, these overexpressed proinflammatory factors in obesity are considered the link between obesity and systemic inflammation (Ellulu et al. 2017). Some clinical studies explore positive associations between different measures of obesity and plasma cytokines, particularly IL-6 levels (Straub et al. 2000), finding that one-third of the total circulating concentrations of this cytokine is derived from adipose tissue (Fontana et al. 2007). In consequence, obesity is regarded as a state of chronic low-grade systemic inflammation (Kim and Nam 2020). Thus, this chronic inflamed state impairs physiological-related tissue and organs function and is correlated with the development of metabolic diseases such as cardiovascular diseases, type 2 diabetes, and hypertension (Castro et al. 2017; Ellulu et al. 2017; Kim and Nam 2020), but also is associated with neurologic disorders such as epilepsy (Daniels et al. 2009; Lee et al. 2011), narcolepsy (Inocente et al. 2013), migraine (Kristoffersen et al. 2020), depression, anxiety, and anhedonia (Dutheil et al. 2016).

### Obesity-Related Chronic Inflammation, and CNS Immune Response

Obesity-induced inflammation is not limited to adipose tissue; many studies relate obesity and its consequences to CNS inflammation (Bruce-Keller et al. 2009). States of overweight and obesity modify the intestinal microbiota, increasing the ratio of *firmicutes/bacteroides*, which has been associated with the increase of free circulating bacterial-derived LPS levels in the blood (Sarmiento-Andrade et al. 2022). As discussed, LPS can activate macrophages and microglia through TLR4 or scavenger receptors, shifting them to a proinflammatory profile (Orihuela et al. 2016; Butovsky and Weiner 2018; Jurga et al. 2020).

Hormones secreted by the WAT can cross the BBB through specialized selective transport systems, but cytokines and chemokines cannot diffuse through them (Banks 2015). However, BBB transport suffers pathological changes during obesity that may exacerbate neurological diseases and lead to peripheral cytokines exposition and immune cell infiltration to the CNS, promoting neuroinflammation and cognitive impairment (Rhea et al. 2017). Thaler et al. suggest that hypothalamic inflammation is the first stage of CNS inflammation since free fatty acids (FFA) and proinflammatory cytokines increase the permeability of the BBB in both human and rodent models (Thaler et al. 2012). Nevertheless, the obesity-induced neuroinflammatory state also involves impairment of other specific brain regions, such as the hippocampus, cerebral cortex, thalamus, putamen, and globus pallidus, among others, as seen in clinical and preclinical studies (Hao et al. 2016; Kim et al. 2020; Gómez-Apo et al. 2021). Animal models show how metabolic compromise relates to neuroimmune activation by increasing proinflammatory chemokines, altering endothelial receptors expression, and diffusion of cytokines that only contribute to a permanent systemic and neuroinflammatory state (Maldonado-Ruiz et al. 2017).

### High-Fat Diet-Induced Obesity Enhances Neuroinflammation and Primes Microglia

Human epidemiological studies have shown a significant positive relationship between the overweight population and fat intake (Lissner and Heitmann 1995). These associations have also been shown in animal studies by inducing obesity with fatty diets, particularly in rodents (Hariri and Thibault 2010). In HFD-fed rats, increased BBB permeability was accompanied by changes in claudin-5, claudin-12, and occluding in the capillaries (Kanoski et al. 2010; Rhea et al. 2017). Ouyang et al. identified more than 45 downregulated proteins in microvessels from obese C57/BL6 mice when compared to chow-fed mice, including proteins involved in cell metabolism, cytoskeleton, cycle regulation, chaperones, scaffolding adaptors, and transport-related mediators (Ouyang et al. 2014), suggesting a direct influence of HFD on BBB integrity and function disruption (Reviewed by Rhea et al. 2017). Proinflammatory molecules and uncontrolled hormone levels can directly affect the brain’s immune responses by altering BBB selectivity (Rhea et al. 2017).

Studies in HFD animal models show the impact of glucose metabolism impairments over the dorsal vagal complex (DVC), where the responses to leptin in DVC glia showed that astrocytes and microglia mediate the anorexigenic effects of leptin in lean rats but not in HFD-induced obese rats (Stein et al. 2020). Microglia and astrocytes of the DVC respond to leptin and loss of energy balance in vivo since the application of leptin decreases astrogliosis in the DVC in HFD mice. In addition, microglia leads to neuroinflammation positively correlated with obesity, supported by its participation in the activation and proliferation of hypothalamic glial cells with the subsequent release of inflammatory cytokines in the hypothalamus (Stein et al. 2020).

The hippocampus, a brain region with a high content of microglia (Lawson et al. 1992), is also influenced by HFD-induced neuroinflammation (de Paula et al. 2021). In this region, microglial activation changes the spatial relationships between microglial processes and synaptic points (Hao et al. 2016). A reduced density of dendritic spines was observed in the granular areas of the dentate gyrus and the CA1 pyramidal areas, and dendritic spines loss was present in regions with increased activation of microglia and phagocytosis of apparently synaptic elements (Cope et al. 2018). These results also showed that inhibiting microglial phagocytic activity with annexin-v improved cognition in obese mice, highlighting the critical role of microglia in cognitive decline during obesity (Cope et al. 2018). In the hypothalamus, previous studies show that overnutrition activates the IKK*β*/NF-*κ*B inflammatory axis and activates proinflammatory signals, leading to microglial activation and developing NF-kB-dependent microgliosis (Zhang et al. 2008).

Incessant exposure of microglia to a proinflammatory environment may lead to their priming and exacerbated response to a future secondary inflammatory trigger (Fernández-Arjona et al. 2022), as discussed previously. Multiple studies have evidenced morphological changes in microglia after feeding animals with HFD (Spencer et al. 2019; Butler et al. 2020; Milanova et al. 2021). In a study with old rats fed HFD for three days, the treatment was sufficient to cause neuroinflammatory differences in the hippocampus and amygdala of the animals. The relevant changes observed in the hippocampal region showed a significant increase in the number of microglial cells and enlarged synaptophysin buttons, evidencing neurodegeneration (Spencer et al. 2019). HFD can also contribute to activating genes involved in this phenomenon, such as CD11b, MHC-II, CX3CR1, NLRP3, and IL-1β in the hippocampus and amygdala, favoring the inflammatory environment that contributes to the neurodegeneration observed in obesity (Butler et al. 2020). As discussed previously, different brain regions are affected by HFD; for example, in the hypothalamus, where the earliest activation occurs due to obesity (from day 1), the microglia lose their phagocytic capacity, while in the hippocampus, the expression of IBA1, CD45, CD68, MHC-II, and IL-1*β* increases (Milanova et al. 2021). This phenomenon shows that microglia are already in an inflammatory profile. Also, microglial activation markers increase in response to HFD and FFA administration, particularly palmitate, in obese rats, suggesting an increased microglia sensitivity and a potentially exaggerated inflammatory response secondary to this exposition (Butler et al. 2020). Another study with C57BL/6 mice fed with HFD for four weeks exhibited decreased hippocampal neurogenesis and elevated IBA1^+^ microglial activation in the ventral hippocampus with increased cytoskeletal protein doublecortin (DCX^+^) inclusions, suggesting an aberrant engulfing capacity. These findings correlate with increased depression and anxiety-like behaviors in HFD-fed mice compared to healthy mice (Yao et al. 2022). Also, the hippocampus was affected in 39 days of HFD-fed mice, in which neuroinflammation was evidenced by raised IBA1^+^ cells reactivity, elevated IL-1*β* and TNF*α* levels that negatively impact spatial memory, cognition, and synaptic plasticity (Vinuesa et al. 2019). Altogether, these observations suggest that microglia cells are also primed by obesity.

These observations correlate with findings in humans patients and animal models confirming that being overweight or obese worsens the development of neurological disorders such as autism spectrum disorder (Trujillo Villarreal et al. 2021), pain (Song et al. 2017; Eichwald and Talbot 2020), epilepsy and seizures (Daniels et al. 2009; Chen et al. 2021), multiple sclerosis (Stampanoni Bassi et al. 2020), bipolar disorders (McWhinney et al. 2021), and depression (Jorm et al. 2003; Dutheil et al. 2016), among others, possibly via microglial priming, as described with aging, CNS trauma, and neurodegenerative diseases (Norden et al. 2015; Li et al. 2018). So, obesity-related low-grade systemic chronic inflammation can prime microglia and promote an exacerbated secondary response to an acute insult, such as CNS trauma or cerebral ischemia.

## Cerebral Ischemia and Microglial Polarization

Stroke is a common disease, defined as a neurological deficit attributed to an acute focal injury of the CNS by a vascular cause (Campbell and Khatri 2020), also considered the second highest cause of death globally and a leading cause of disability in adults, with one in four people affected over their lifetime worldwide (Campbell et al. 2019). Strokes are classified as either ischemic or hemorrhagic. Ischemic stroke is responsible for most cases, about 85%, against 15% of hemorrhagic (Hinkle and Guanci 2007). Among ischemic stroke variants, ischemic due to reduced blood flow resulting from arterial occlusion is more common than venous infarction due to occlusion of cerebral veins or venous sinuses (Campbell and Khatri 2020). Some epidemiological studies suggest that approximately 90% of strokes are attributable to modifiable risk factors, including blood pressure, smoking, diabetes, hyperlipidemia, physical inactivity, and obesity (O’Donnell et al. 2010; Pandian et al. 2018).

Most ischemic strokes are due to extracranial and intracranial artery embolisms when the blood flow is affected by the narrowing of vessels, reducing brain blood flow, causing severe stress and sudden cell death by necrosis. This event precedes a disruption of the plasma membrane, organelle swelling, and leaking of cellular contents into the extracellular space accompanied by energy failure, increased extracellular calcium levels, penumbral neurons excitotoxicity, oxidative stress, infiltration of leukocytes and microglia-mediated neuroinflammation (Kuriakose and Xiao 2020). When an ischemic event happens, collateral blood flow supply can maintain the viability of hibernating brain regions around the injury site for a limited time, in which microglia and astrocytes try to exert salvage mechanisms; this region is the ischemic penumbra (Campbell et al. 2019).

### Microglial Role During Cerebral Ischemia Development and Recovery

**Fig. 3:**
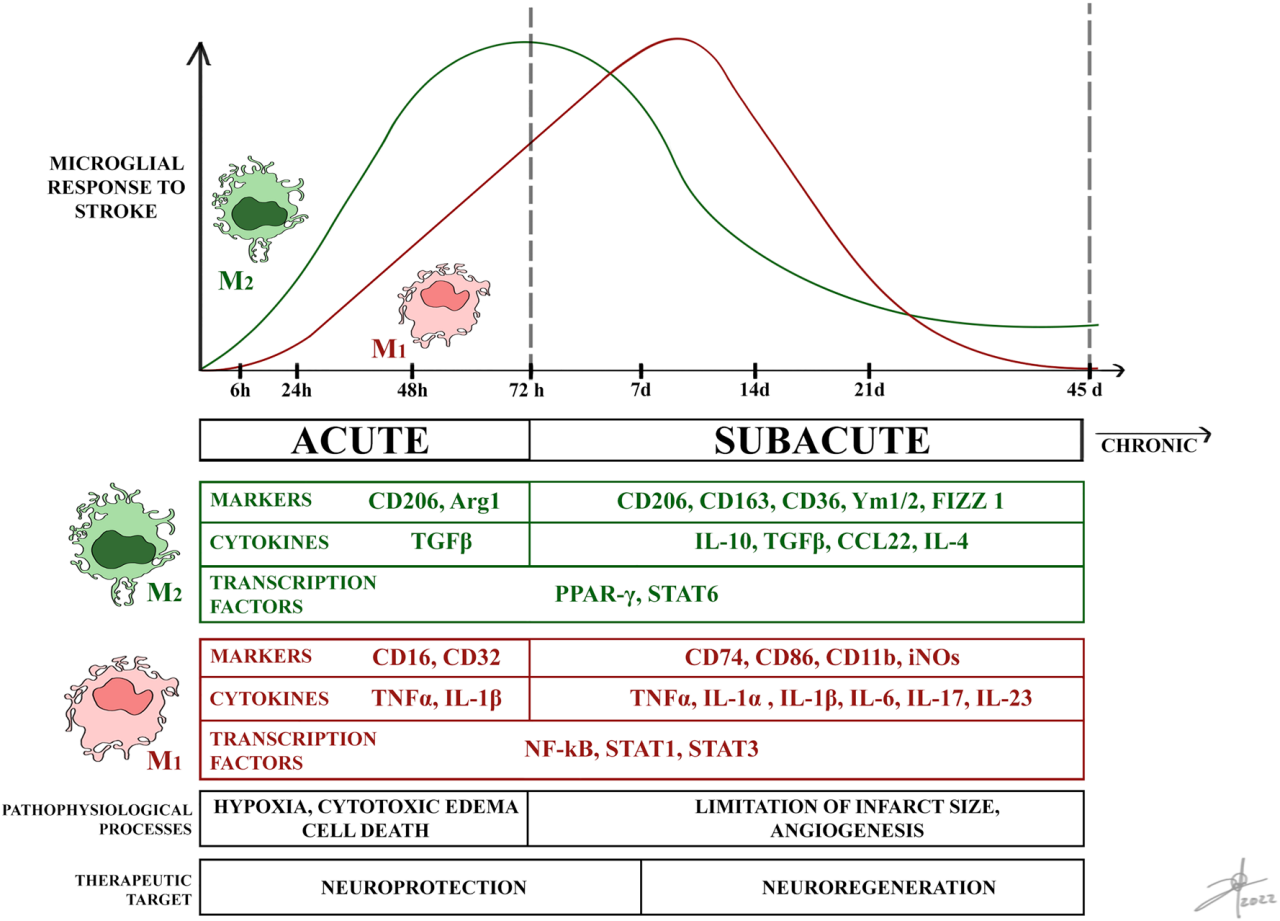
Changes in the microglial phenotype in the development of the pathophysiology of cerebral stroke. The infarct window presented shows the acute and subacute stages. Surface markers, cytokines secreted by microglial phenotypes, and transcription factors that induce modifications in the microglial phenotype stand out, in addition to the treatment target according to the temporality of the disease. Abbreviations: cluster of differentiation (CD), arginase 1 (Arg1), transforming growth factor β (TGFβ), inducible nitric oxide synthase (iNOs), interleukin (IL), peroxisome proliferator-activated receptor gamma (PPAR-*γ*), signal transducer and activator of transcription (STAT), nuclear factor kappa B (NF-κB).

Microglia is the first cell in response to pathophysiological changes produced by cerebral infarction, migrating immediately towards the lesion site, producing proinflammatory cytokines and cytotoxic substances while also contributing to tissue repair and remodeling by cleaning cellular debris and secreting anti-inflammatory cytokines and neurotrophic factors, exerting a dual role (Qin et al. 2019). The temporality of the pathophysiology in cerebral infarction is divided into the acute period (hours), where excitotoxicity damage prevails; subacute (days and weeks), in which damage secondary to inflammation appears; and chronic (months), in which long-term damage establishes (Fig. 3) (Dirnagl et al. 1999). Following ischemia, microglia and recruited macrophages are activated and present different phenotypes and morphologies, which change during these time lapses (Qin et al. 2019). The IBA1 marker begins to rise at approximately 3.5 h and continues to rise steadily until 24 h, showing its peak at four to seven days, keeping a more ramified morphology between days one to three and more ameboid on day six (Kang et al. 2020). Ameboid microglia can present an M_1_ phenotype producing proinflammatory mediators including TNF*α*, IL-6, IL-1*β*, IFN*γ*, iNOS, and proteolytic enzymes such as metalloproteinases MMP-9 and -3 (Yenari et al. 2010), but also the M_2_ phenotype is present during acute phases. The subtype M_2*a*_ favors cellular regeneration, both M_2*b*_ and M_2*c*_ are involved in phagocytosis and elimination of necrotic tissue, and all are characterized by the production of IL-10, TGF*β*, IGF-1, and VEGF, which are anti-inflammatory and pro-angiogenic (Qin et al. 2019).

Cerebral ischemia has a dual microglial response depending on the stage of brain injury, finding proinflammatory and anti-inflammatory actions in the acute and resolving course of infarction. Microglia cells detect extracellular signals, perform transduction for gene expression, and activate both responses (Dong et al. 2021). In the acute phase, a predominance of M_2_ reaches its maximum peak before the first week, preceded by M_1_, which has its maximum peak in the second week (Zhang et al. 2022). Also, at 72 h post-infarct, more peripheral macrophages are present than active microglia, which predominantly secrete IL-1*β* and have greater TNF*α*-dependent phagocytic capacity (Ritzel et al. 2015). Studies performed to differentiate the microglial population from infiltrating peripheral macrophages show that TMEM119 is a valuable marker for distinguishing these two populations in developing cerebral infarction (Young et al. 2021). The phagocytic function is essential in the recovery from cerebral infarction. Transcriptomic studies in models of middle cerebral artery occlusion (MCAO) in Cx3cr1^−^CreER^+/−^; Nhe1^flox/flox^ (Nhe1 cKO) mice, demonstrated that the KO of NHE1, an essential regulator of aerobic glycolysis in microglia, directly affects the phagocytic functions of these cells resulting in increased microglial phagocytic capacity and helps neuronal remodeling, improving cognitive function after infarction (Song et al. 2022).

During subacute and chronic phases, microglia respond to localized damage and participate in the neurodegeneration secondary to infarction into the penumbral zone, which involves the progressive loss of tissue nearby to the primary infarction (Dirnagl et al. 1999). In 2019, Kluge et al. performed a spatiotemporal analysis of microglia secondary to stroke in the cortex and thalamus on day three to 56 post-injury with Cx3CR1 GFP/WT mice, using a photothrombotic occlusion model. They established the time course and the regional specificity of impairment and found that microglia at the peri-infarct site extended their processes out towards the local damage at all along the time course and acquired an M_1_ phenotype, correlating NeuN loss with morphological microglia disturbances, with a peak of microglial activation and neuronal degeneration 28 days after stroke (Kluge et al. 2019).

Microglia phenotype M_2_ exerts a neuroprotective effect by modulating cell function; M_2_ microglia-derived exosomes were able in MCAO models to reduce infarct injury, attenuate behavioral deficits, and in vitro improve neuronal survival following direct exosome-mediated cell interaction (Song et al. 2019). The exosome mechanisms of the M_2_ phenotype are currently an important target of study; several authors have studied them in models other than cerebral infarction, such as TBI, demonstrating that this interaction via the exosome leads to the growth of neurites and decreases inflammation (Huang et al. 2018). In these pathways of extracellular vesicles of Microglia M_2_, the action of neurotrophic factor TGF*β* has been highlighted as an activator of the Smad2/3 pathway in endothelial cells and neurons, which contributes to the regulation of an early post-infarction inflammatory response that leads to the improvement of individuals at the behavioral level by reducing neuronal death (Zhang et al. 2022). These studies suggest that all the diversity of microglial function is vital for the recovery from cerebral infarction. Nevertheless, microglial response in cerebral ischemia has been studied under previous physiological healthy states. The emerging question is: Can obesity-primed microglia worsen ischemic stroke patients?

## Can Obesity-Induced Primed Microglia Exacerbate Cerebral Ischemia Damage?

Among the world's leading risk causes of stroke are elevated systolic blood pressure, poor diet that includes fast food diet, high body mass index, high LDL cholesterol, high fasting glucose, and low physical activity (Feigin et al. 2022).

Obesity is a separate risk factor that has been linked to previous and even after-infarction complications. Clinical research has shown the link between systemic inflammation and the beginning of myocardial infarction or cerebral ischemia; however, the data are contradictory. Some studies, as published by Bauza et al. 2018, monitored patients for up to a year after the infarction. Obese and diabetic individuals presented no differences in mortality or disability evident after the infarction compared to healthy patients (Bauza et al. 2018), even though other researchers had demonstrated that the waist-to-hip ratio (WHR) was a predictor of the occurrence of a stroke (Aparicio et al. 2019). Nevertheless, Bauza and their colleagues mentioned the need to perform new analyses to differentiate between metabolically healthy patients from metabolically unhealthy patients within the obesity BMI categories to clarify the possible mechanisms by which obesity and diabetes may interact with ischemic stroke (Bauza et al. 2018).

Most of the risk factors for stroke are modifiable, and their management improves the incidence of stroke. Hyperlipidemia evaluation and treatment is a critical part of stroke management, as mentioned by the American College of Cardiology in 2013, providing a perspective on statin treatment, improving the cholesterol profile by 30% to 50%, and lowering the risk for stroke (Stone et al. 2014). Also, metabolic syndrome and disorders of glucose metabolism are major risk factors for stroke, being highly prevalent in patients with stroke, associated with a 60% risk in diabetic patients and 22% in metabolic syndrome US population (Guzik and Bushnell 2017). Although obesity is considered a well-established risk factor for ischemic stroke, epidemiological studies are controversial, showing that BMI correlates with improving or worsening stroke incidence and mortality (Quiñones-Ossa et al. 2021). Gruberg et al. coined the term “Obesity paradox” for the counterintuitive finding that obese patients had better outcomes than normal-weight patients, all with coronary artery disease (Gruberg et al. 2002). The obesity paradox has also been documented in other cardiovascular diseases, including unstable angina, myocardial infarction, coronary artery bypass graft, chronic heart failure, and percutaneous coronary intervention (Forlivesi et al. 2021). Nonetheless, in stroke, the relationship between obesity and mortality remains controversial.

Most epidemiological studies on the obese population measure BMI to segment groups for the analyses, but it has been criticized because it does not differentiate among fatness, obesity, and adiposity measurement, so all obtained information can be wrongly classified and generate controversial results (Quiñones-Ossa et al. 2021). Individuals are classified according to their BMI into five categories: underweight (BMI < 18.5 kg/m^2^), normal weight (BMI 18.5–24.9 kg/m^2^), class I obesity—overweight (BMI 25.0–29.9 kg/m^2^), class II obesity—obesity (BMI 30.0–39.9 kg/m^2^), class III obesity—extreme obesity (BMI > 40 kg/m^2^) (De Lorenzo et al. 2016). Also, a sustained increase in weight gain in the industrialized population promoted the need for a superior obesity class for morbid obesity, establishing class IV obesity as 50 to 59.9 kg/m^2^ and class V BMI > 60 kg/m^2^ (Elagizi et al. 2018). However, these values are not a measurement of adiposity, only an imprecise mathematical estimate. Some authors reported a U-shaped relationship between BMI and stroke mortality, where the lowest mortality risk was found in BMI around 35 kg/m^2^, and the highest with BMI < 31 kg/m^2^ and > 38 kg/m^2^ (Forlivesi et al. 2021), but this classification remains controversial because of lack information from the metabolic and inflammatory states of these obese people, highlighting the pathogenic role of adipose tissue, termed “adiposopathy” (De Lorenzo et al. 2016). Adiposopathy is associated with adverse endocrine and immune responses leading to metabolic disease, knowing that not all patients who are obese have a metabolic disease, and not all patients with metabolic diseases are obese or overweight (Bays and Ballantyne 2006). So other metabolic-related obesity classifications are needed because of the endocrine dysfunction and low-grade chronic inflammation roles, which can promote re-analyses of controversial studies to compare them better. In addition, adiposopathy and general obesity were found as potential risk factors for worsening different pathologic conditions (Bruce-Keller et al. 2009).

Aging studies have shown that aging-primed microglia increased mRNA and protein expression of various inflammatory markers and alterations in morphology, such as MHC-II and CD11b, in both rodents and post-mortem aged human brain studies (Frank et al. 2006; Norden and Godbout 2013), in addition to the increase of basal levels of TNFα, IL-1β, and IL-6, all proinflammatory cytokines, and NLRP3 inflammasome (Youm et al. 2013). In another context, TBI causes prolonged microglial activation in the lesion border, promoting a primed state of activation, which means a higher expression of proinflammatory mediators, including MHC-II, CD68, and NOX2 in the thalamus and cortex 12 months after injury (Loane et al. 2014). Prion and AD pathologies induce microglial activation and priming to a basal proinflammatory profile, increasing the expression of immune markers, such as MHCI-II, CD68, TREM2, and NLRP3, which contribute to neurodegeneration, promoting a sustained inflammation in the brain’s parenchyma by neurodegeneration-primed microglia (Cameron and Landreth 2010; Cunningham 2013; Norden et al. 2015). At last, severe or prolonged sepsis promotes repeated stimulation to microglia in the CNS via systemic LPS and its consequent systemic inflammation, which makes microglia more sensible to consecutive LPS challenges, increasing the expression of cytokines as IL-1*β*, TNF*α*, and IL-6 (Cunningham 2013). What do all these pathological conditions have in common? Every condition primed microglia and promoted to respond vigorously to subsequent inflammatory stimulation, evolving from an adaptive local CNS inflammatory response to a chronic or exacerbated proinflammatory response, enhancing damage secondary to the stimulus (Cameron and Landreth 2010; Cunningham 2013; Norden et al. 2015). Hence, can obesity be a synergic condition to microglial activation and promote exacerbated secondary damage to neuropathologies? Some obesity-induced low-grade systemic chronic inflammation studies showed that obesity could be a potential precondition to induce chronic neuroinflammation and enhance proinflammatory microglial responses in neuropathologies, translating into worsened prognostic. HFD-induced obese mice for 20 weeks exposed to a PD model showed an increased number of IBA1 + microglia in the substantia nigra and striatum compared with controls correlating with a significant impairment in motor coordination and reduction of dopaminergic neurons (Kao et al. 2020). Another study in rats aimed to determine the neuroinflammatory consequences of eight weeks of HFD before animals suffer chronic unpredictable mild stress. Experimental results showed a synergistic effect of HFD-induced obesity with neuroinflammation developed by stress, demonstrated with overexpressed IL-1*β*, IL-6, and TNF*α* levels in the hippocampus compared to alone HFD or mild stress treatment, which associates with an increase in depression-like behavior in rats (Wang et al. 2022). So obese-primed microglia can induce a secondary enhanced proinflammatory response to neuropathologic events.

What happens if a stroke is the second challenge to primed microglia? During aging, primed microglia express higher levels of interferon regulatory factor (IRF)5 and lower levels of IRF4; these proteins regulate an inflammatory axis during stroke induced by middle cerebral artery occlusion (MCAO), which comes into detrimental effects in mice, also validated with its transgenic knockout IRF4 and IRF5 variants (Ngwa et al. 2022). This IRF5-IRF4 axis forms a regulatory signaling pathway to balance microglial pro- and anti-inflammatory activation (Al Mamun et al. 2020). Aged-primed and IRF4-KO microglia enhance IL-1*β* and TNF*α* production during MCAO, which correlates with a significantly larger infarct volume in the cortex and the whole ipsilateral hemisphere (Ngwa et al. 2022). In another context, recent studies showed that acute ischemic stroke is more prevalent in COVID-19 patients than in non-infected populations. This risk increases when comorbidities such as hyperlipidemia, diabetes, or hypertension are present in patients before the stroke (Qureshi et al. 2021). This event is associated with the COVID-19 microglia hyperactivation, known as the “two-hit” hypothesis, where severe COVID-19 patients carry pre-activated microglia, which may be due to cytokine storm and gets an exaggerated response during a second challenge (Bouayed and Bohn 2021). Some animal studies have shown a possible interaction between obesity, microgliosis, and exacerbated damage in a gerbil model of forebrain ischemia; nevertheless, its mechanisms were not fully cleared (Song et al. 2018; Seo et al. 2020). It is possible that obese-primed microglia can enhance the secondary-stroke damage in the CNS, similarly as demonstrated in aging and COVID-19 contexts.

## Conclusions

Even though obesity-related mechanisms for enhancing neurological pathologies are not fully understood, experimental and human studies have shown that obesity and overweight are associated with low-grade systemic chronic inflammation, and microglia-directed proinflammatory responses (Bruce-Keller et al. 2009; Wang and Li 2021), highlighting the exacerbated response of primed microglia induced by chronic inflammation, as discussed before. So, obesity-primed microglia can be the primary responsible for enhancing stroke damage when patients are metabolically sick, overweight, or obese.

## Data Availability

Not applicable.
